# Supplementary figures and images for: Agrobacterium-mediated transient transformation of sorghum leaves for accelerating functional genomics and genome editing studies
Source: BMC Res Notes. 2020 Feb 27;13:116. doi: 10.1186/s13104-020-04968-9 (PMC7045639; doi:10.1186/s13104-020-04968-9)

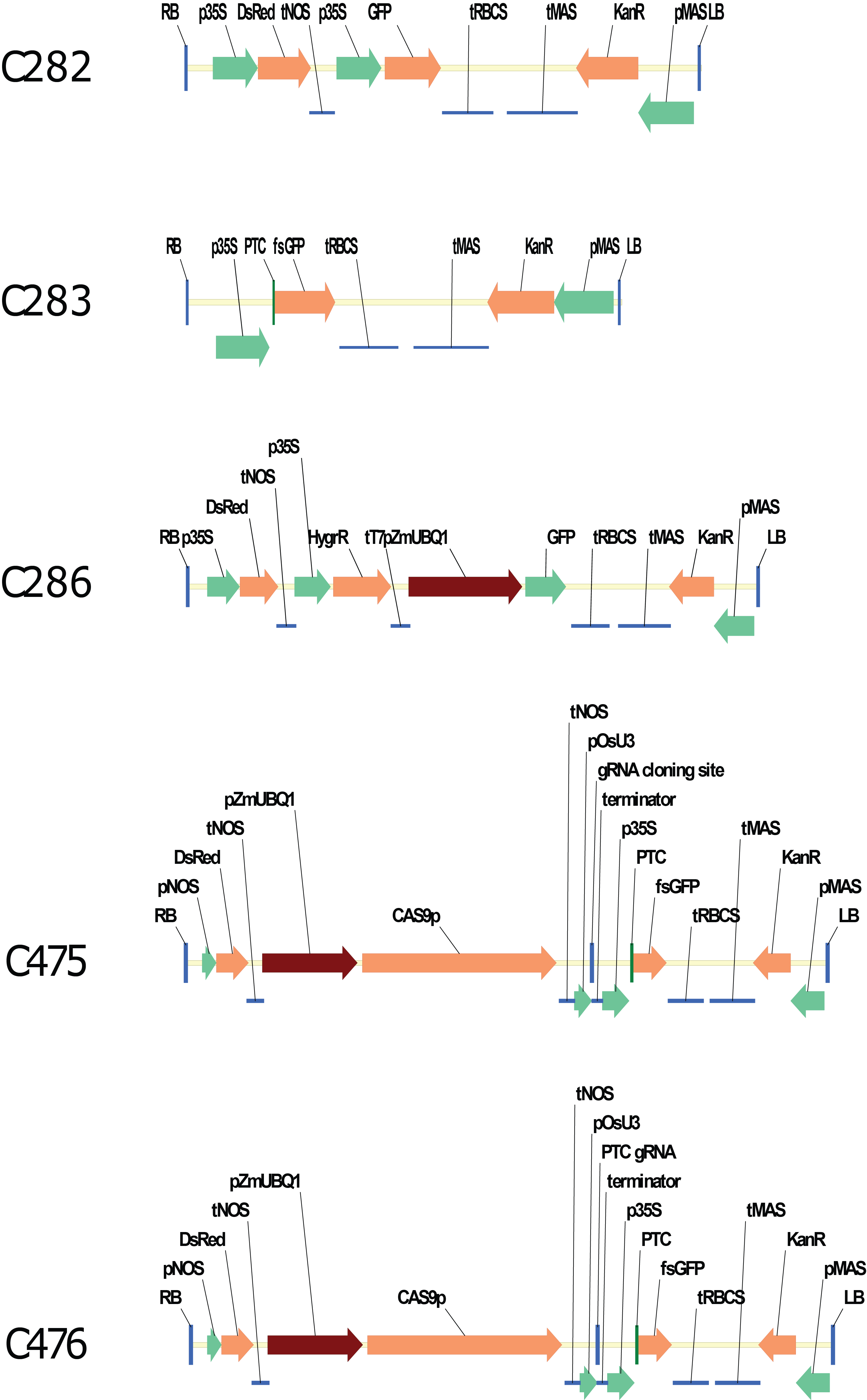

Supplement: Supplementary file 1 — Additional file 1: Figure S1. Schematic presentations of the T-DNA regions of transformation constructs. Elements in each construct are drawn to scale. LB, left border of the T-DNA region; RB, right border of the T-DNA region; PTC, positive target control site for genome editing; fsGFP, frame-shifted GFP with PTC inserted after ATG start codon [file 13104_2020_4968_MOESM1_ESM.png]
